# LC Passive Wireless Sensor System Based on Two Switches for Detection of Triple Parameters

**DOI:** 10.3390/s22083024

**Published:** 2022-04-14

**Authors:** Muhammad Mustafa, Mian Rizwan, Muhammad Kashif, Tahir Khan, Muhammad Waseem, Andres Annuk

**Affiliations:** 1 Micro/Nano Systems Integration R&D Centre, School of Microelectronics, University of Science and Technology of China, Hefei 230026, China; mustafaktk@mail.ustc.edu.cn; 2 School of Electrical Engineering, Southeast University, Nanjing 210096, China; rizwan.nazeer26@uog.edu.pk (M.R.); kashifktk@seu.edu.cn (M.K.); 3 School of Electrical Engineering, Zhejiang University, Hangzhou 310027, China; tahirkhan@zju.edu.cn; 4 Chair of Energy Application Engineering, Institute of Forestry and Engineering, Estonian University of Life Sciences, 51006 Tartu, Estonia; andres.annuk@emu.ee

**Keywords:** inductive coupling, multifunction, mechanical switches, passive wireless sensing system

## Abstract

This paper presents the LC-type passive wireless sensing system for the simultaneous and independent detection of triple parameters, featuring three different capacitive sensors controlled by two mechanical switches. The sensor coil was connected with three different capacitors in parallel and two mechanical switches were in series between every two capacitors, which made the whole system have three resonant frequencies. The readout coil was magnetically coupled with the sensor coil to interrogate the sensor wirelessly. The circuit was simulated advanced design system (ADS) software, and the LC sensor system was mathematically analyzed by MATLAB. Results showed that the proposed LC sensing system could test three different capacitive sensors by detecting three different resonant frequencies. The sensitivity of sensors could be determined by the capacitance calculated from the detected resonant frequencies, and the resolution of capacitance was 0.1 PF and 0.2 PF when using the proposed sensor system in practical applications. To validate the proposed scheme, a PCB inductor and three variable capacitors were constructed with two mechanical switches to realize the desired system. Experimental results closely verified the simulation outputs.

## 1. Introduction

A square spiral inductor is coupled to a sensing capacitor to generate a resonant LC circuit in the inductor-capacitor (LC) sensor. The variable capacitor varies in response to the parameter of interest that causes the readout coil impedance or input impedance to detect a resonant frequency when change occur. Passive wireless LC sensors are frequently employed in applications where monitoring external parameters such as pressure [1,2], temperature [3,4], chemical concentration [5], and air humidity [6,7] is problematic due to a lack of a physical link. Small size, low cost, contactless interrogation, and an unlimited lifetime are all advantages of LC sensors [8,9]. Passive wireless sensing technology has been utilized to monitor several other characteristics besides non-contact detection via LC wireless sensors [10]. Practical implementations in the sensor network required the detection of multiple parameters independently and simultaneously [11].

As a result, in a variety of applications, the LC passive wireless sensor system for multi-parameter measurement is needed. The most straightforward way to design a multifunctional LC sensor is to create an array of LC circuits with separate LC resonant circuits responding to the many parameters of interest; however, this results in a larger sensor chip area and readout coil size. Quality factor and resonant frequency are measured using the temperature effect of an LC passive wireless sensor to monitor the two parameters of pH value and temperature [12]. Another approach for measuring temperature and humidity was used to monitor the resonant frequency and real magnitude value of maximum input impedance [13]; however, the method was limited due to the LC sensor’s operating principle. Using two inductors can solve this issue; however, because of the significant mutual coupling between the two inductors, the transmission signals interfere with each other, and the resonant frequencies are altered or absent entirely. Both capacitive sensors were used with embedded inductors to measure the temperature and humidity resulting in a limited interrogation range [14]. Another attempt was made to employ particular winding stacked inductors, which presented several LC tanks to realize multi-parameter measurements [15], but the interrogation range was limited due to the sensor’s relatively low mutual inductance. The use of a relay switch to control two capacitive sensors has been proposed in a double parameter measuring approach [16]. The two resonant frequencies corresponding to the two sensors were detected simultaneously and independently; however, the sensitivity of the sensors identified was limited in double parameter detection, and their size was sufficient in comparison to the multiple parameters needed. Strong magnetic coupling occurs when the sweep frequency matches the resonant frequencies simultaneously and independently, allowing the sensor inductor to receive maximum energy from the readout coil [17,18].

Wireless low-power sensor systems are attractive in many new applications because they can transmit data and energy wirelessly while requiring no physical connection between the sensor and the processing units. These systems are extremely reliable in tough situations. Wireless sensors can operate in two modes: active and passive. The derivation of active sensors is from internal battery source, while passive sensors are charged by an inductive system. Active sensors have several drawbacks, including a longer readout distance, extra installation and maintenance, cost, and battery life [19].

For simultaneous detection of multi-parameters, different approaches have been used in various practical applications such as implantable devices, food sample monitoring used in critical situations about patients [20,21,22], tooth enamel detection [23], humidity measurement of sealed packages [24,25], and so on. The advanced research on inductor-capacitor sensors in recent years has rapidly increased and the growing variety of applications necessitates the use of multi-parameters. An array of separated inductor capacitor sensors, on the other hand, would take up a lot of space and require a separate readout coil for each sensor [26,27,28] in addition to other limitations.

**Figure 1:**
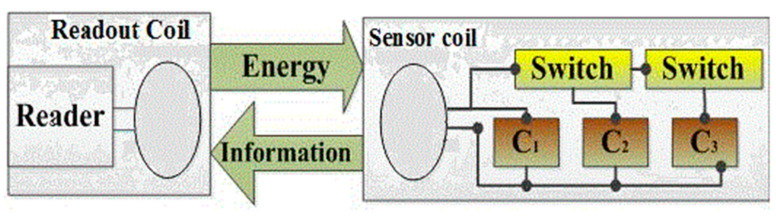
Scheme of LC passive wireless sensor inductive link.

The sensor inductor induces voltage for sensor operation when readout coil is magnetically coupled, and control of the switches in this novel structure of multifunctional LC passive wireless sensing system consists of one inductor, three variable capacitors, and two switches, which construct three different LC tanks. The three different resonant frequencies respond simultaneously to three different variable capacitors. Figure 1 shows the model of the proposed system. In Section 2, the resonant frequencies are analyzed by Kirchhoff’s law of the proposed system and simulations using advanced designing system (ADS-2016) to demonstrate the resonant frequencies. In Section 3, the experimental setup and results are presented. Finally, the conclusion is given in Section 4.

## 2. Operation Principle

### 2.1. Analysis

**Figure 2:**
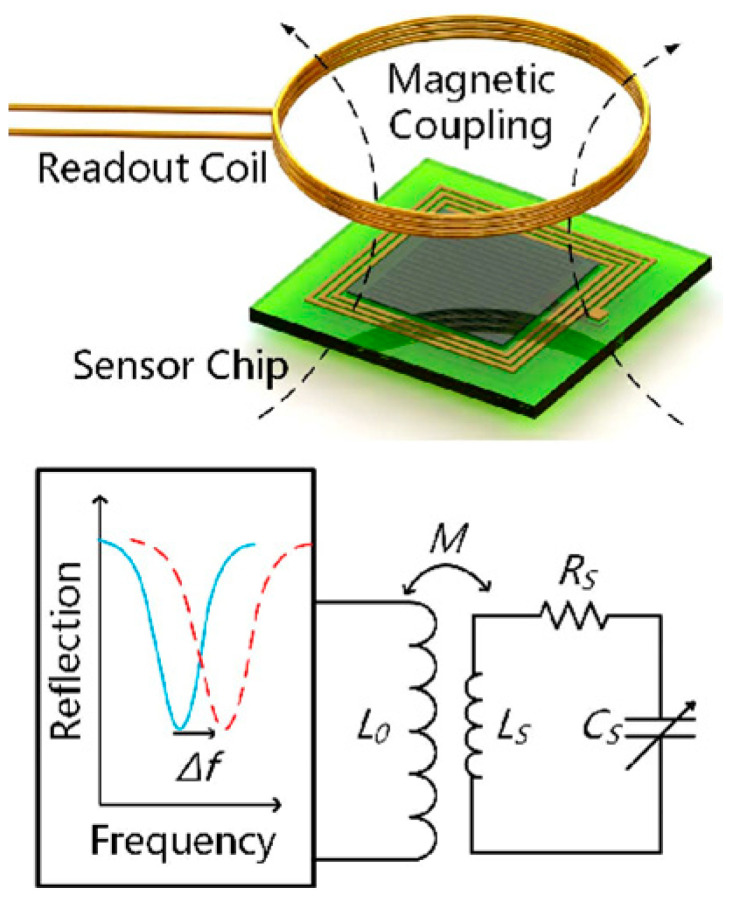
Graphical representation of LC passive wireless sensor system.

The LC sensing behavior is explained in Figure 2. For wireless interrogation of the LC sensor, used readout coil was magnetically connected with a sensor coil, and the sensor’s resonant frequency was measured in response to the parameter sensed.

The resonant frequency is given by:

(1) $$f=\frac{1}{2\pi \sqrt{\mathrm{LC}}}$$

The geometry, distance between the readout and sensor coil, and all inductive coupling influences in the magnetic medium is shown in Figure 2 [29]. The simplified equation for coupling coefficient k is calculated as follows:

(2) $$k=\frac{1}{{\left[1+2^{\frac{2}{3}}{\left(\frac{x_{12}}{\surd r_{1}r_{2}}\right)}^{2}\right]}^{\frac{3}{2}}}$$

where $r_{1}$ and $r_{2}$ are the radii of the area enclosed by both inductors which are 10 mm each and $x_{12}$ represents the distance between the coils which is 6 mm. The value of k lies between 0 and ±1, where ±1 means the maximum coupling between two coils and 0 means no coupling. The value of k for the proposed model was 0.6 according to the Equation (2). The coupling can be improved by optimizing the size of the coils and the distance between them.

Figure 2 depicts the reader coil and sensor coil of the LC passive wireless sensor system, with the reader coil having 2.5 loops and the sensors inductor consisting of 10 turns of planar square loops. The inductance value of the sensor inductor was 0.5 µH. The sensor inductor had an outer diameter of 10 mm and a line width of 150 µm, and 100 µm was the distance between the two loops. The thickness of copper was 30 µm.

The readout and sensor coils were magnetically coupled to each other with mutual inductances. The readout coil worked as a transducer to transmit the power to the sensor and receive a signal in the form of resonant frequency to measure the sensor changes.

**Figure 3:**
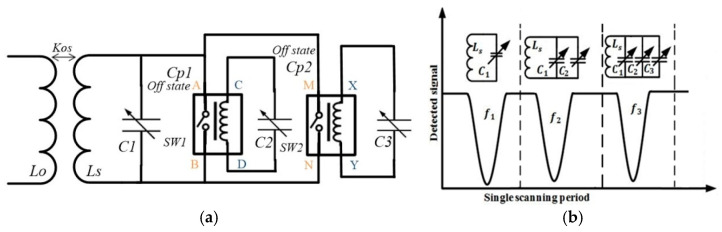
Theoretical model demonstration (**a**) LC triple parameters monitoring system integrated with two relay switches. (**b**) Representation of working principle.

Both of the switches consisted of four ports as shown in Figure 3a; two ports both were voltage ports, A and B for switch ${\mathrm{Sw}}_{1}$, and M, N for ${\mathrm{Sw}}_{2}$, while the other blue points C, D, X, and Y were the electrical connection ports, respectively. The readout coil induced alternating voltages into the sensor inductor by magnetic coupling, and the mechanical switches were mechanically controlled. The ${\mathrm{Sw}}_{1}$ turned on when the voltage reached the threshold, which made an electrical connection between capacitor $C_{1}$ and $C_{2}$ parallel.

At the start of the scanning cycle, the capacitor $C_{1}$ represented a resonant circuit with the sensor inductor $L_{s}$ exhibiting parallel connection, as shown in Figure 3b, where the first resonant frequency $f_{1}$ was detected by the readout coil ($L_{o}$), as well as the power coupled by sensor coil $L_{s}$ from the readout coil $L_{o}$ to the mechanical switches. Turning on ${\mathrm{Sw}}_{1}$ connected $C_{2}$ with the capacitor $C_{1}$ in parallel to form the second resonant circuit with the sensor inductor to detect $f_{2}$. When $f_{2}$ was consistent with the scanning frequency, the induced voltage approached the threshold of the switch ${\mathrm{Sw}}_{2}$, and we turned on the switch ${\mathrm{Sw}}_{2}$. Meanwhile, the switch ${\mathrm{Sw}}_{1}$ turned off because the scanning frequency increased and mismatched with $f_{1}$. The capacitor $C_{3}$ became connected with the $C_{1}$ and $C_{2}$ in parallel to represent the third resonant circuit, and readout coil was used for the detection of the third resonant frequency $f_{3}$. Finally, the controlling voltage approached the level where it was refused by both switches and turned off. The main factor here was the voltage interval to keep both the switches turned on and to ensure that the switches were not be turned off until the sweeping frequencies captured the detected frequencies ($f_{1}$, $f_{2},$ and $f_{3}$) consequently. Another important consideration was the turn-off delay time of both switches, which should be in the tens of milliseconds range to ensure that the detected frequencies $f_{2}$ and $f_{3}$ are recorded by the sweeping frequencies. The readout device performed the frequency scanning operation automatically, and changing the sweep frequency band controlled the sweep intervals among the detected frequencies ($f_{1}$, $f_{2}$, and $f_{3}$). When the scanning cycle was completed, the entire circuit returned to its initial condition and was prepared for the next cycle. The detected resonant frequencies ($f_{1}$, $f_{2}$ and $f_{3}$) could be defined as:

(3) $$f_{1}=\frac{1}{2\pi \sqrt{\mathrm{Ls}\left(C_{1}+C_{\mathrm{CD}}+\frac{C_{p1}\cdot C_{2}}{C_{p1}+C_{2}}+C_{\mathrm{XY}}+\frac{C_{p2}\cdot C_{3}}{C_{p2}+C_{3}}\right)}}$$

(4) $$f_{2}=\frac{1}{2\pi \sqrt{\mathrm{Ls}\left(C_{1}+C_{\mathrm{CD}}+C_{2}+C_{\mathrm{XY}}+\frac{C_{p2}\cdot C_{3}}{C_{p2}+C_{3}}\right)}}$$

And

(5) $$f_{3}=\frac{1}{2\pi \sqrt{\mathrm{Ls}\left(C_{1}+C_{\mathrm{CD}}+C_{2}+C_{\mathrm{XY}}+C_{3}\right)}}$$

where $C_{p1}$ and $C_{p2}$ represent the parasitic capacitances because both switches during off-state are connected in series with capacitors $C_{2}$ and $C_{3}$ in practical applications. The $C_{\mathrm{CD}}$ and $C_{\mathrm{XY}}$ are also parasitic capacitances across both switches, which had an insignificant effect but needed to be considered during simulations. The effect of both switches’ resistance was equivalently varied with the capacitors in parallel, which had the same parasitic effect of capacitors as discussed above. In this case, the Equations (3)–(5) were simplified and appropriate. Both switches had measured resistance of 1.53 ohms during off-states, which had little impact on the detected resonant frequencies. The above three equations were used for the calculation of resonant frequencies of three capacitors, while parasitic capacitances of both switches are given in datasheets and also were tested through LCR meter.

### 2.2. Evaluation and Comparison

The comparison and description of different multi-parameter LC sensor systems are given in Table 1 below. The proposed model had excellent contributions in different aspects. The proposed scheme had higher measuring sensitivity, minimum cost, lower chip area by utilizing two mechanical micro switches, larger interrogating range of about 6 mm, detected and measured three parameters independently, and had negligible parasitic capacitance effect as compare with the other existing approaches.

### 2.3. Simulations

**Figure 4:**
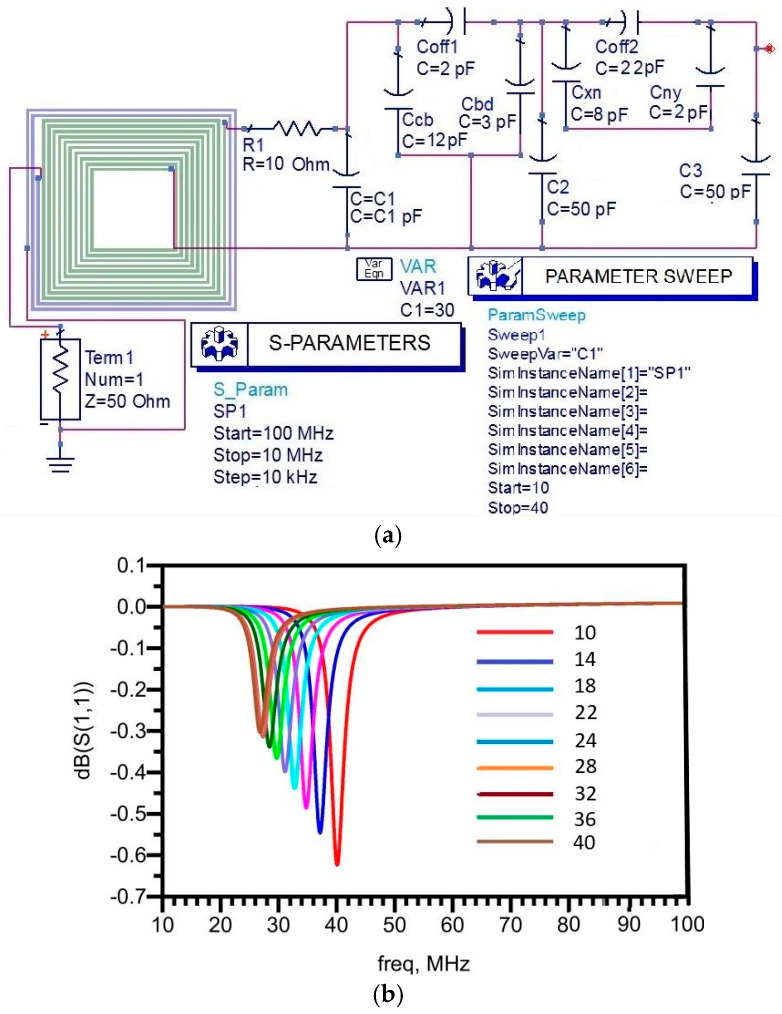
Layout simulations setup using ADS and results. (**a**) Simulation setup when both switches were in off-state. (**b**) Detected resonant frequencies by applying sweep parameter at $C_{1}$.

According to the mathematical analysis, $f_{1}$ > $f_{2}$ and $f_{2}$ > $f_{3}$ or $f_{1}$ > $f_{3}$. The resonant frequency simulation using ADS in three different and individual setups is given in Figure 4a, which shows the first detected resonant frequency for a more distinct analysis of comparison among the three resonant frequencies and three capacitors. The readout coil’s inductance was commonly assumed to be 1 µH, while the sensor coil’s inductance was 0.5 µH, and three capacitors were independently set from 10 pF to 200 pF. At off-state, the parasitic capacitance of the first switch ${\mathrm{Sw}}_{1}$ was $C_{p1}$ of 18.6 pF, while the parasitic capacitance of the second switch $C_{p2}$ was 45 pF. When the variable capacitance $C_{1}$ was changed but $C_{2}$ and $C_{3}$ were kept constant at 50 pF, the observed resonant frequencies are shown in Figure 4b where both switches were turned off when the sweep parameter was applied to the variable capacitance $C_{1}$. 

**Figure 5:**
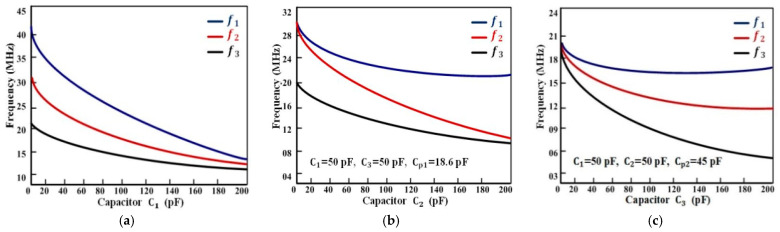
Simulation curves of resonant frequencies versus three capacitors, respectively (**a**) Resonant frequencies versus $C_{1}$ when $C_{2}$ and $C_{3}$ were fixed at 50 pF; (**b**) resonant frequencies versus $C_{2}$ when $C_{1}$ and $C_{3}$ were fixed at 50 pF; (**c**) resonant frequencies versus $C_{3}\;$ when $C_{1}$ and $C_{2}$ were fixed at 50 pF.

Figure 5a shows that the three resonant frequencies decreased gradually when $C_{1}$ increased and $f_{1}$, $f_{2}$, and $f_{3}$ became close to overlapping each other as $C_{1}$ was much larger than $C_{2}$ and $C_{3}$. This condition made it difficult to solve the capacitances. By turning on the first switch ${\mathrm{Sw}}_{1}$, the capacitor $C_{2}$ changed but $C_{1}$ and $C_{3}$ were fixed at 50 pF. The difference between the detected resonant frequencies $f_{1}$ and $f_{2}$ enlarged when $C_{2}$ became much larger than $C_{1}$, but the difference between $f_{2}$ and $f_{3}$ decreased to become overlapped with each other as $C_{2}$ was much larger than $C_{3}$, as shown in Figure 5b.

Similarly, when both switches were turned on, $C_{3}$ changed but $C_{1}$ and $C_{3}$ were fixed at 50 pF, then the difference among three resonant frequencies increased as much as $C_{3}$ increased, and $f_{3}$ could still decrease greatly but ${\;f}_{1}$ and $f_{2}$ kept unchanged, as shown in Figure 5c. These differences among the resonant frequencies occurred because of the parasitic capacitances $C_{p1}$ and $C_{p2}$, which were connected in series with the capacitors $C_{2}$ and $C_{3}$, consequently weakening the influence of change in the detected resonance frequencies.

## 3. Experiments and Results

**Figure 6:**
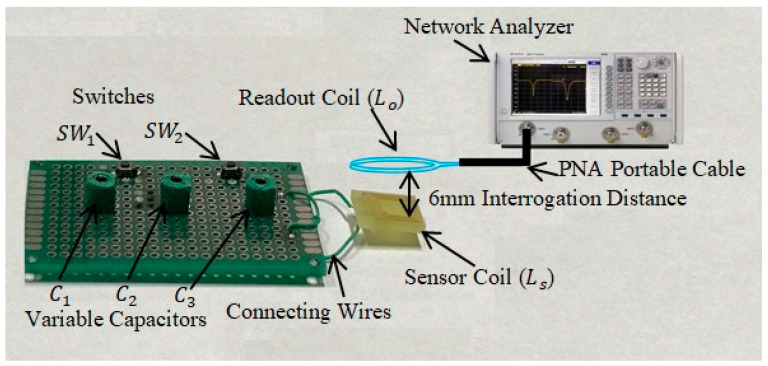
Schematic diagram of experimental setup.

To verify the proposed multifunctional sensor system, the three variable capacitors were tested by controlling two mechanical switches instead of electromagnetic relay switches. The schematic diagram of the experimental setup of LC triple parameter sensor system designed and constructed on the PCB board is shown in Figure 6. The sensor inductor $L_{s}$ was used to receive the energy by mutual coupling and transmitted the signal to capacitors. The three variable capacitors $C_{1}$, $C_{2}$ and $C_{3}$ were connected in parallel and with the two switches ${\;\mathrm{Sw}}_{1}$ and ${\mathrm{Sw}}_{2}$, respectively. The variable capacitors imitated three capacitive sensors to monitor different parameters by detecting resonant frequencies at the readout coil. The mechanical switches were used to control the circuit. However, by using an electromagnetic relay switch, the threshold voltage for one switch was 10 V and for second switch was 20 V, as available commercially. The tested parasitic capacitance of mechanical switch ${\mathrm{Sw}}_{1}$ in the off-state was 0.96 pF and for ${\mathrm{Sw}}_{2}$ was 0.65 pF, which could be neglected to be considered during simulation, but the parasitic capacitances $C_{p1}$ of 18.6 pF and $C_{p2}$ of 45 pF were connected in series with $C_{2}$ and $C_{3}$ in practical applications when both the switches were in the off-state.

**Figure 7:**
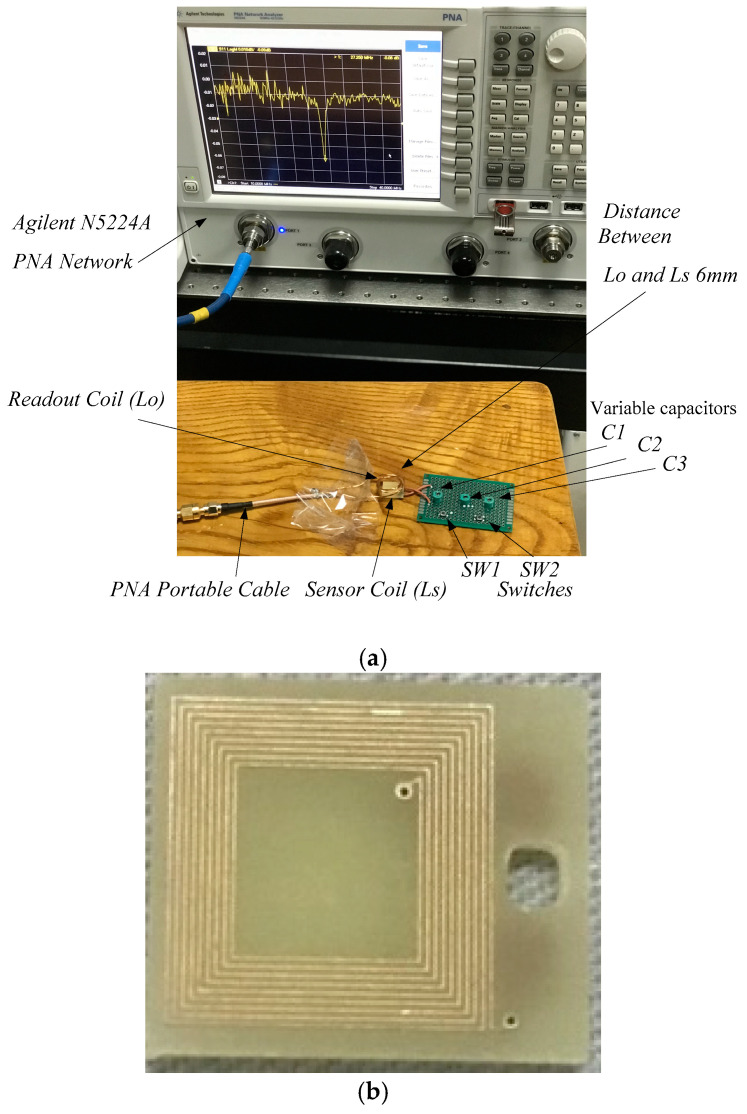
Experiments of the LC sensor system. (**a**) Experimental platform of readout coil and monitoring sensor system. (**b**) PCB planar square copper inductor.

The actual fabrication and testing was accomplished as depicted in the experimental setup Figure 7a. As a sensor inductor, a PCB planar square copper inductor was employed, which was integrated over a PCB with 10 turns and 0.5 µH inductance, as illustrated in Figure 7b. Outer diameter of sensor inductor was 10 mm, and the line’s width was 150 µm. The distance between loops was 100 µm. The copper had a thickness of 30 µm, while the PCB substrate was 2 mm thick. The sensor coil inductance was 0.5 µH.

The LC triple parameters monitoring sensor system and readout coil were connected to a network analyzer and the distance between the readout coils was fixed at 6 mm. The diameter was 10 mm and was coupled to an Agilent N5224A PNA network analyzer (PNA) to monitor the LC tank’s frequency response. When the PNA’s output power was set to 20 dBm, the distance between the readout coil and sensor inductor was modified to evaluate the system’s mutual coupling capability and switch operation. 

**Figure 8:**
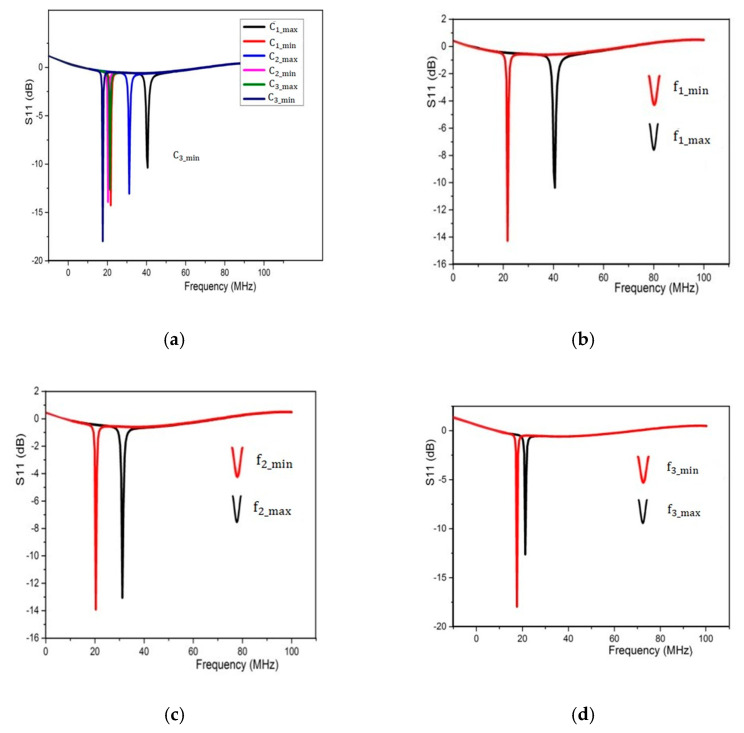
Experimental results of detected resonant frequency versus S11 parameter of proposed LC sensor system, (**a**) combined representation of detected frequencies for maximum and minimum values of three capacitors, (**b**) curves for variation in $C_{1}$, (**c**) curves for variation in $C_{2}$, (**d**) curves for variation in $C_{3}$.

The Figure 8a shows different resonant frequencies detected by changing the values of variable capacitors one by one.

When both of the switches $({\mathrm{Sw}}_{1}$ and ${\mathrm{Sw}}_{2})$ were in the off-state and $C_{1}$ measured at a minimum value of 14.6 pF and the tested parasitic capacitances of both mechanical switches were 0.96 pF for ${\mathrm{Sw}}_{1}$ and 0.65 pF for ${\mathrm{Sw}}_{2}$, then the maximum resonant frequency $f_{1\_\max}$ was detected as 40.6 MHz, as shown in Figure 8b. Similarly, the minimum detected resonant frequency $\left(f_{1{\_}_{\min}}\right)$ was 21.7 MHz by measuring the variable capacitor $C_{1}$ at a maximum value of 44.6 pF.

By turning on the first switch ${\mathrm{Sw}}_{1}$, the variable capacitor $C_{2}$ was set to a minimum value of 8.73 pF, while $C_{1}$ was at a maximum of 44.6 pF. The second resonant frequency $f_{2}$ varied from 30.7 MHz to 20.35, as shown in Figure 8c. The capacitance of $C_{2}$ changed from 44.6 pF to 47 pF.

Figure 8d shows the third resonant frequency $f_{3\_\max}$ of 21 MHz when both switches were turned on and the variable capacitor $C_{3}$ was measured at a minimum value of 20 pF, but $C_{1}$ and $C_{2}$ were fixed at 44.6 pF and 47 pF. respectively. The third resonant frequency $f_{3}$ varied from 21 MHz to 17.65 MHz and the capacitance of $C_{3}$ changed from 20 pF to 40.2 pF, while $C_{1}$ and $C_{2}$ were fixed at 44.6 pF and 47 pF with their respective parasitic capacitances of 18.6 pF and 45 pF connected in series with them, which had to be considered during use in practical applications.

We can see in Figure 8c,d that the resonant frequencies decreased, the influence of the parasitic capacitance connected in series with both of the capacitors, which greatly weakened the influence of changing capacitances and resulted in the small change in resonant frequencies.

The theoretical analysis of three capacitors was calculated by Equations (3)–(5) when the three resonant frequencies were given by the readout coil of the LC sensor system. When the $C_{1}$ is increased more than the other two capacitors, the three frequencies became close or even overlapped, which was already explained in Section 2 in detail and represented in Figure 5a. In this situation, the proposed LC sensor system could not be applied in real applications. Hence, the position of the capacitor $C_{1}$ and $C_{2}$ were exchanged for the solution of overlapping of the three resonant frequencies to each other, but still, ${\;f}_{2}$ and $f_{3}$ overlapped, as shown in Figure 5b. Therefore, positions of the two capacitors were also exchanged which addressed the merging issue of the three resonant frequencies, then we could calculate the three capacitors by using the three equations. The equivalent circuit was already discussed in the theoretical model which was simplified and had the small parasitic capacitances at the other ports of both switches taken into consideration. The resistance affected the Q factor of the whole LC circuit; however, it had a small effect over the resonant frequency. The final result was not influenced by the resistance. 

## 4. Conclusions and Future Work

In conclusion, an LC-type passive wireless triple parameter monitoring system with double mechanical switches was designed and manufactured. The corresponding circuit mentioned in the theoretical model was simplified and did not account for any other considerations, particularly the resistance and minor parasitic capacitances at both switches’ off-state ports. The resistance had an impact on the overall Q factor of the LC circuit, but only a little impact on the resonant frequency. As a result, the lack of resistance had no bearing on the ultimate results. Two factors influenced the coupling voltage: the distance between the readout coil and the sensor inductor and the difference between the sweep frequency and the LC tank’s resonance frequency. The schematic circuit of the multi-parameter monitoring LC sensor system was simulated using ADS software and the mathematical analysis was carried out using MATLAB 2010. The experiments showed that the two mechanical switches were controlled and actuated mechanically to demonstrate and verify the detected resonant frequencies for three parameters. It was shown from the measurements that three different resonant frequencies could respond to the three variable capacitors, respectively. Therefore, the proposed multi-parameter LC sensor system could measure three parameters. In the future, further techniques and methodologies can be utilized to achieve the issue of multiple sensor operation simultaneously through a miniaturized and limited-area-acquired inductive telemetry system.

## Figures and Tables

**Table 1:** Comparison of different multi-parameter approaches’ limitations and aspects.

Approaches	Number of Parameters	Limitations	Inducement
Two quantities of detected resonant frequency signal	Two	Lower measuring sensitivity, lower signal strength, lower accuracy	Working principle
Array of inductors capacitor circuit	Two	Larger chip area, higher cost, lower measuring sensitivity, signal strength	Individual resonant circuits
Stacked inductors circuits	Two	Transmitting signal shifts, detected signal missing or shift, higher cost, lower measuring sensitivity	Strong mutual coupling
Two partly overlapped inductors system	Two	Limited measuring range, lower signal strength, lower measuring sensitivity, higher cost	Interrogation distance
Specific winding stacked inductors system	Two	Limited measuring sensitivity, lower signal strength, higher cost, limited interrogating distance	Small mutual inductance
Two parallel LC circuits using single relay switch	Two	Limited measuring sensitivity, limited signal strength, higher cost, larger chip area, distorted detected signal, limited interrogating range	Relay switch
